# Chlorhexidine gluconate usage is associated with antiseptic tolerance in staphylococci from the neonatal intensive care unit

**DOI:** 10.1093/jacamr/dlab173

**Published:** 2021-11-17

**Authors:** Dheeraj K Sethi, Heather Felgate, Maria Diaz, Kirstin Faust, Cemsid Kiy, Paul Clarke, Christoph Härtel, Jan Rupp, Mark A Webber

**Affiliations:** 1 Quadram Institute Bioscience, Norwich Research Park, Norwich NR4 7UQ, UK; 2 Norwich Medical School, University of East Anglia (UEA), Norwich, UK; 3 Department of Pediatrics, University of Lübeck, Lübeck, Germany; 4 Neonatal Unit, Norfolk and Norwich University Hospital (NNUH), Norwich NR4 7UY, UK; 5 Department of Pediatrics, University of Würzburg, Würzburg, Germany; 6 Department of Infectious Diseases and Microbiology, University of Lübeck, Lübeck, Germany

## Abstract

**Background:**

Intravascular catheters are essential for care in Neonatal Intensive Care Units (NICUs) but predispose infants to catheter-associated infections including late-onset sepsis, commonly caused by CoNS. Antiseptics are applied to prevent infection with chlorhexidine (CHG) and octenidine (OCT) the most common agents used.

**Objectives:**

To investigate the association between antiseptic use and bacterial susceptibility.

**Methods:**

CoNS isolates were collected from two NICUs with differing antiseptic regimens: Norwich, UK (using CHG) and Lubeck, Germany (using OCT). CoNS were isolated from different body sites of babies upon admission, and weekly thereafter. Antiseptic susceptibility testing was performed, and a selection underwent genome sequencing.

**Results:**

A total of 1274 isolates were collected. UK isolates (*n = *863) were significantly less susceptible than German isolates (*n = *411) to both CHG (mean MIC: 20.1 mg/L versus 8.9 mg/L) and OCT (mean MIC: 2.3 mg/L versus 1.6 mg/L). UK isolates taken on admission were more susceptible to CHG than subsequent isolates. No cross-resistance between the agents was seen. Genome sequencing of 122 CoNS showed the most common species to be *Staphylococcus epidermidis* and *Staphylococcus haemolyticus* and phylogenetic analysis suggested antiseptic tolerance evolved multiple times in independent lineages. There was no evidence of dominant antiseptic tolerant clones and carriage of genes previously implicated in antimicrobial susceptibility (*qac*, *smr*, *norA*/*B*), did not correlate with CHG or OCT susceptibility.

**Conclusions:**

Long-term CHG use may select for CHG and OCT tolerance in CoNS. This highlights the different potential for separate antiseptic regimens to select for resistance development. This could be an important factor in developing future infection control policies.

## Introduction

Preterm neonates (born <37 weeks gestation) are predisposed to infection and often require intravascular catheterization, which breaches skin and provides an access route for pathogens.^1^ Late-onset sepsis (LOS) refers to bloodstream infections seen >72 h after birth and is most commonly caused by CoNS, with *Staphylococcus epidermidis* accounting for 57% to 78% of LOS in developed countries.^2–6^ For infants weighing <1500 g, LOS is associated with mortality of 17% to 19%,^7^^,^^8^ and can cause significant neurodevelopmental disability.^9^^,^^10^ Once regarded as innocuous commensals, CoNS are now better recognized as important opportunistic pathogens.^11^

The source of infection in LOS remains controversial. Although there is evidence of bacterial translocation from the gut mucosa,^12^ colonization of indwelling vascular catheters is regarded as a major cause of infection. The ability of CoNS to form biofilms on indwelling devices is important,^13^^,^^14^ and catheters can be inoculated by skin-dwelling CoNS upon insertion, leading to catheter-related infections.^15^^,^^16^

Strict adherence to aseptic protocols is a crucial preventative measure with antiseptics being used prior to line insertion to reduce infection risk.^17^^,^^18^ This is associated with a reduced risk of endogenous contamination from the skin microbiota.^19–21^ Among the most common antiseptics used on neonates are chlorhexidine gluconate (hereafter referred to simply as ‘chlorhexidine’; CHG) and octenidine dihydrochloride (hereafter referred to simply as ‘octenidine’; OCT). Both are cationic surfactants with bactericidal and bacteriostatic properties, however despite their widespread usage, mechanisms of action and resistance remain unclear.^22–24^

There is marked heterogeneity in neonatal antiseptic practices, and no national guidelines available in the UK. Virtually all units utilize antiseptics pre-procedurally (i.e. before intravascular catheter insertion), to reduce numbers of organisms on skin and sometimes to bathe neonates, though practices vary widely.^25–27^ Antiseptic use in neonates is complicated by uncertain safety profiles, consequently, weaker antiseptic concentrations are used in this group than adults. This presents a theoretical risk of sub-optimal antimicrobial efficacy, and the potential for selection of resistant mutants.^28^

A survey of UK Neonatal Intensive Care Unit (NICU) antiseptic practices showed chlorhexidine is used in 56 of 57 units. Chlorhexidine at 0.015% with 0.15% cetrimide is used in seven units, whilst chlorhexidine-exclusive compounds range in concentration from 0.05% to 2%, with both aqueous and alcoholic preparations seen.^19^ In Germany, octenidine is predominantly used with both alcoholic and aqueous preparations used, and 0.1% octenidine is used by >95% of units.^20^ There is no consensus regarding which antiseptic is most appropriate for neonates, and whether alcoholic or aqueous preparations should be preferred.^27^^,^^28^

Previously, we studied the susceptibility of a population of *Staphylococcus aureus* to chlorhexidine and octenidine. These isolates were from adult patients in a single UK hospital-trust over a period where antiseptic usage changed. We identified a correlation between the emergence of isolates demonstrating decreased susceptibility to chlorhexidine and octenidine, related to usage. The clinical implications and mechanisms of this adaptation are unclear,^24^ and the question of whether antiseptic use might aid in the selection and expansion of antiseptic-tolerant clones in the clinical environment remains unanswered. However, there is evidence strains with decreased chlorhexidine susceptibility thrive in environments where chlorhexidine is used.^29^

Given the long-term use of antiseptics at low concentrations in NICUs, the selection of strains with reduced antiseptic susceptibility represents a real concern. To investigate whether antiseptic usage influences susceptibility in CoNS, we studied CoNS from NICUs with differing antisepsis protocols. The Norfolk and Norwich University Hospital’s NICU (UK) has used chlorhexidine for over 20 years. Concentrations in use range from 0.015% to 2% and octenidine is not routinely used. The Lübeck NICU (Germany) has exclusively used 0.1% octenidine since 2013.

Both units engaged in a prospective study, collecting staphylococcal isolates from infants on admission into the unit and weekly thereafter, over a 3 month period. Susceptibility of isolates to chlorhexidine and octenidine and biofilm formation were determined. A selection of isolates underwent genome-sequencing to investigate the phylogenetic relationships between isolates and mechanisms of antiseptic resistance. Similar strain types were present in both locations, chlorhexidine usage was associated with reduced susceptibility to both antiseptics, and this was seen in multiple lineages and species. No clonal expansion was identified, and resistance did not correlate with known mechanisms of antiseptic tolerance.

## Materials and methods

### Study sites

Both the Norfolk and Norwich University Hospital’s NICU in the UK and the NICU in Lübeck, Germany provide Level 3 medical and surgical care.

### Isolate collection

Infants admitted to NICU over a 10 week period were enrolled. Each NICU had routine MRSA surveillance policies that involved taking skin swabs from all infants upon admission and weekly thereafter. For the present study, duplicate swabs were taken and positive clinical cultures were also included, during the study period.

For the UK unit, enrolment was between November 2017 and January 2018. Skin isolates were collected using Amies Charcoal Swabs (Sterillin^TM^, Thermo Fisher Scientific, USA) from all infants on admission and weekly until discharge. Swabs from the ear, nose, groin and rectum are taken as part of routine MRSA surveillance. Additional, duplicate swabs were taken for isolation of CoNS in this study. These were plated on 5% horse blood agar (Oxoid, Thermo Fisher Scientific, USA), prior to incubation at 37°C for 24 h. Colonies with a morphology consistent with CoNS were sub-cultured onto mannitol-salt agar (Oxoid, Thermo Fisher Scientific, USA) and coagulase tests performed. Isolates were stored on preservation beads at −80°C (Protect, Technical Service Consultants Ltd, UK) and in 96 deep-well plates in 20% glycerol at −40°C.

In the German unit, infants weighing <1500 g in the unit during the study period (January to March 2018) were enrolled. Swabs were taken using Amies Charcoal Swabs (Hain Lifescience GmbH, Germany), from the axilla, rectum and groin. Colonies identified morphologically as staphylococci underwent MALDI-TOF mass spectrometry (Bruker, USA), prior to preservation at −80°C on beads (Microbank™, Thermo Fisher Scientific, USA) and transportation to the UK.

### Antimicrobial susceptibility testing

Susceptibility to antiseptics and antibiotics [benzylpenicillin, cefotaxime, vancomycin, gentamicin, fusidic acid and daptomycin (for daptomycin, media were supplemented with Ca^2+^ to give a final concentration of 50 mg/L)] was determined for the isolates that subsequently underwent whole-genome sequencing. This was performed using agar dilution, following EUCAST guidelines (www.eucast.org). Doubling serial dilutions of either antiseptic in Mueller–Hinton agar (Sigma–Aldrich, USA) were prepared and inoculated with 10^4^ cfu of each isolate using a 96-pin replicator. Inoculated antimicrobial plates were incubated at 37°C for 24 h. Control strains *Staphylococcus aureus* ST 239 (TW20) and *S. aureus* NCTC 8532 (F77) were used.^29^ The correlation between chlorhexidine or octenidine and each antibiotic was assessed with linear regression using GraphPad Prism 7 (v7.04).

### Biofilm quantification

Biofilm quantification used a crystal violet assay in duplicate. Overnight cultures of each isolate were prepared in 200 mL of tryptic soy broth with 1% glucose (Sigma–Aldrich, USA). Each 96-well plate was washed to remove planktonic cells and incubated in 0.1% crystal violet for 10 min. This plate was washed again, prior to the addition of 70% ethanol to solubilize crystal violet retained by adherent biofilm. Absorption at 590 nm was measured for each well using a FLUOstar^®^ Omega plate reader (BMG Labtech, UK).

### DNA extraction

Isolates were selected to include the highest and lowest levels of antiseptic susceptibility, both study sites and all body sites. Isolates acquired longitudinally from the same babies were also included. Isolates were grown in 1 mL of Mueller–Hinton broth overnight in 96 deep-well plates and incubated overnight at 37°C. Cells were harvested by centrifugation at 3500 **g** and resuspended in 100 μL of lysis buffer (5 mg/L lysozyme, 20 mg/L lysostaphin, 0.1 mg/mL RNAse in Tris-EDTA, pH 8). Suspensions were transferred to a semi-skirted, low-bind PCR plate, secured with an adhesive seal, and incubated at 37°C with agitation at 160 rpm for 25 min. Cells were lysed by addition of 10 μL of lysis buffer (50 g/L SDS, 1 mg/mL proteinase K, 1139 mg/mL RNAse in Tris–EDTA, pH 8) and incubated at 65°C at 1600 rpm for 25 min, after this the plate was sealed with PCR strip lids. The plate was briefly centrifuged and 100 μL of the lysed cells were transferred to new PCR plates. To isolate DNA, 50 μL of DNA-binding magnetic beads (KAPA Pure beads, Roche diagnostics) were added to the samples and incubated at room temperature for 5 min. The plate was placed on a magnetic base and the supernatant removed by pipetting. Beads were washed three times with 80% ethanol and left to air dry for 2 min. The DNA was eluted from the beads using 50 μL of 10 mM Tris-Cl, pH 8.5 and removing the samples from the magnetic platform. Finally, the isolated DNA was transferred to a new PCR plate. DNA concentration was determined using the Qubit dsDNA HS Assay 150 kit.

### Whole-genome sequencing

Genomic DNA was normalized to 0.5 ng/μL using EB (10 mM Tris-HCl) and libraries were prepared from 1 ng of DNA by ‘tagmentation’ using the Illumina Tagment DNA Enzyme and Buffer before amplification with the Kap2G Robust PCR kit (Sigma) and the Nextera XT Index Kit v2 index primers (Illumina), following manufacturer’s instructions. Libraries were quantified using the Quant-iT high sensitivity dsDNA Assay Kit and pooled following quantification in equal quantities. The final pool was double-SPRI size-selected between 0.5× and 0.7× bead volumes using KAPA Pure Beads (Roche) and normalized to a final concentration of 1.8 pM. The library was sequenced using an Illumina Nextseq500^TM^ instrument using a Mid Output Flowcell v2 (Illumina).

### Bioinformatic analysis

Raw FASTQ reads were processed using Kraken (v 1.1.1)^30^ to check for contamination. Samples containing non-staphylococcal reads were excluded. Processed reads were then compared with the NCBI RefSeq genomes database using RefSeq Masher (v0.1.1)^31^ and isolates assigned a putative species identification. Samples were grouped by species and the closest reference in RefSeq for each group used as a reference for identifying core SNPs using Snippy (v4.4.3) (*S. epidermidis* NIH04003, accession number GCF_000276005; *S. haemolyticus* C10A, accession number GCF_000764065.1; *S. aureus* subsp. *aureus* 21311, accession number GCF_000626975.1). An alignment of core SNPs was produced using snippy-core and used to build a phylogenomic tree under the best-fit model (GTR+F+R7 for *S. epidermidis*, TVM+F+I+G4 for *S. haemolyticus* and GTR+F+G4 for *S. aureus*) implemented in iq-tree (v1.6.12).^32^ Trees were visualized and metadata added using iTOL (v 5.5.1).^33^ Antimicrobial resistance genes were detected in the processed reads using ARIBA (v2.12.2)^34^ and databases ResFinder (v 4.0) and CARD (v 3.0.1).^35^ Assembled genomes were uploaded onto the NCBI BioProject Database (ID: PRJNA751027).

To identify any links between *norA* and *norB* genotypes and antiseptic tolerance, genomes were assembled using Shovill (v1.0.4).^36^*norA* and *norB* genes were identified in the assembled genomes using nucleotide-nucleotide BLAST (v2.7.1),^37^ and the gene sequences plus 200 bp upstream were extracted using bedtools (v2.27).^38^ The extracted sequences were aligned using clustal (v2.1),^39^ and trees were built based on the alignment using the neighbour-joining method.^40^

### Ethics approval

This study was reviewed by the Research and Development Manager for the Norfolk and Norwich University Hospitals NHS Foundation Trust and approved as a surveillance study, which did not require formal ethics committee review. In Germany, the study was reviewed and approved as a surveillance study by the University of Lubeck Hospital ethics committee (reference AZ 15-034, amendment 01/2018).

## Results

### Isolates and characteristics

A total of 1274 staphylococci were isolated from babies from the two sites over 10 week periods. Of these, 863 isolates were from the UK unit, with 30.8% from the ear (*n = *266), 31.3% from the nose (*n = *270), 22.0% from the groin (*n = *190) and 15.9% from the rectum (*n = *137). 82.6% of isolates (*n = *713) were collected from weekly screening, and 17.4% (*n = *150) from admission swabs. From the German unit, 411 isolates were recovered, 45.7% of these were from the axilla (*n = *188), 32.6% from the groin (*n = *134), 19.0% from the rectum (*n = *78) and 1.5% from the throat (*n = *6). In addition, one isolate was from a blood culture, and two were from wound swabs. Admission isolates comprised 15.2% of the German isolates (*n = *56) with the remainder from weekly swabbing (84.8%).

### Species identification

UK staphylococcal isolates were identified morphologically, by coagulase test results and by growth on mannitol-salt agar. German isolates underwent MALDI-TOF (as part of routine practice in Lübeck) which identified 8 *S. aureus* isolates, whilst the remaining 403 were CoNS. 71.1% of all isolates were *S. epidermidis* (*n = *292), 16.6% *S. haemolyticus* (*n = *68), 6.8% *S. hominis* (*n = *28) and 3.7% *Staphylococcus capitis* (*n = *15).

### Antiseptic susceptibility testing

**Figure 1.**
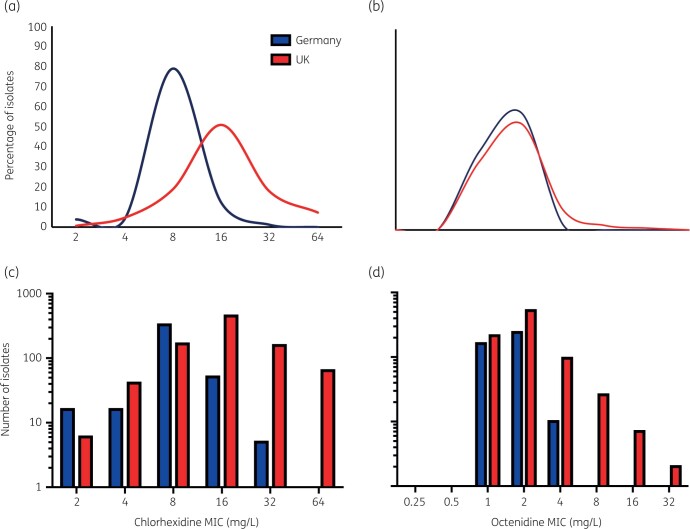
Susceptibility of UK and German isolates to chlorhexidine and octenidine, as a percentage of all isolates from either study site (panels a and b) and as actual numbers (panels c and d). Blue lines or bars represent data from German isolates, red lines and bars isolates from the UK.

UK isolates were significantly less susceptible to chlorhexidine than German isolates (*P < *0.0001 by Chi squared test). The UK isolates’ mean MIC was 20.1 mg/L (range: 2–64 mg/L, MIC_90_ 32 mg/L), versus 8.9 mg/L (range: 2–32 mg/L, MIC_90_ 16 mg/L) for German isolates (Figure 1a, c). All isolates with an MIC of chlorhexidine of 64 mg/L (*n = *64) were from the UK.

**Figure 2.**
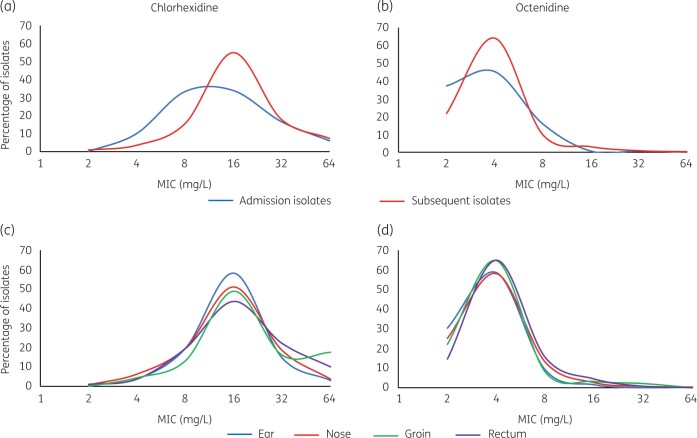
Antiseptic susceptibility of UK isolates from admission and subsequent samples and from different body sites. Panels (a) and (b) show susceptibility to chlorhexidine and octenidine of isolates recovered at admission (blue lines) and from subsequent weekly swabs (red lines). Panels (c) and (d) show susceptibility of all isolates stratified by the body site they were recovered from for each antiseptic.

There was a significant difference (*P < *0.0001) between chlorhexidine susceptibility of isolates taken on admission (mean MIC of 17.7 mg/L) versus weekly screening (mean MIC of 20.6 mg/L) for UK isolates, with admission isolates more susceptible than those collected later (Figure 2a). Susceptibility of isolates was similar across different body sites (Figures 2c and d), except for UK isolates from the groin. Of these, a high proportion exhibited a reduced susceptibility to chlorhexidine (≥32 mg/L) (Figure 2c). No significant differences were seen in chlorhexidine susceptibility of German isolates, when comparing swab site or when isolates were taken.

Comparison of octenidine susceptibility between UK and German isolates also showed a significant difference (*P < *0.0001). Though the mean MICs of octenidine against both populations were similar (2.3 versus 1.6 mg/L, respectively) all the isolates with highest MICs (≥8 mg/L) were from the UK (Figure 1b, d). Once again, UK isolates collected on weekly screening demonstrated reduced octenidine susceptibility, as compared with admission isolates (Figure 2b). No significant differences in octenidine susceptibility according to body site were observed.

Although the UK isolates demonstrated highest MICs of both agents, there was no correlation between susceptibility to octenidine and chlorhexidine when comparing all isolates, or any breakdown by location of body site (Figure S1, available as Supplementary data at *JAC-AMR* Online). For the sequences that underwent whole genome sequencing, further antimicrobial susceptibility testing was performed for a selection of antibiotics. No significant correlation was identified between MICs of chlorhexidine or octenidine and any of the antibiotics tested (Table S1).

### Quantification of biofilm-forming capacity

No significant differences were observed between the biofilm-forming capacity of isolates with regards to their origin location (UK or Germany), whether they were from admission or weekly screening swabs, or the body site they were collected from. A comparison of chlorhexidine and octenidine susceptibility with biofilm formation did not show any correlation.

### Genome sequencing

**Figure 3.**
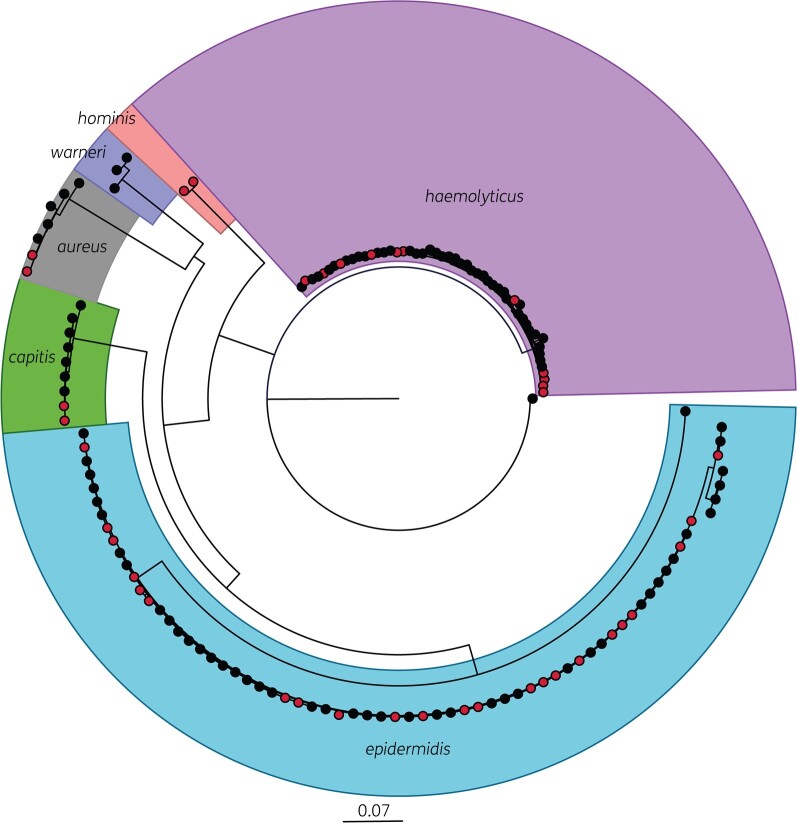
Population structure of sequenced isolates. Phylogenetic tree based on core SNP alignment of isolates with site of isolation indicated (black circles indicate UK isolates, red circles German isolates).

**Figure 4.**
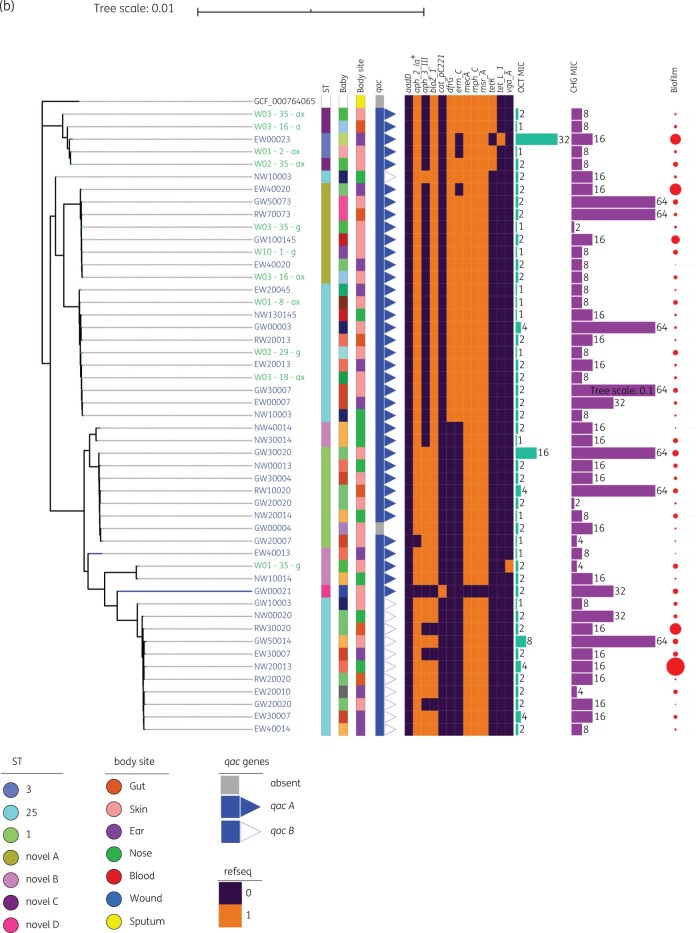
Continued

A selection of isolates with different phenotypes were sequenced, resulting in 122 assembled staphylococcal genomes. *S. epidermidis* accounted for 53.3% of these (*n = *65), *S. haemolyticus* (*n = *49) for 40.1% and of the remaining eight isolates, six were *S. aureus* and two *S. hominis.* A phylogenetic assessment of all sequenced isolates showed no major differences in strain types present in the German and UK NICUs with related strains present in both sites (Figure 3). Analysis of the phylogeny of *S. epidermidis* and *S. haemolyticus* isolates against the body site from which isolates were collected showed no significant correlation (Figure 4a, b), suggesting neonatal CoNS isolates are not highly adapted to different body sites. No relationship was observed between the phylogeny of strains of either species and MICs of chlorhexidine or octenidine. Isolates with elevated chlorhexidine or octenidine MICs did not belong to a single clade, and antiseptic tolerance appears to have evolved on multiple occasions amongst several independent lineages.

**Figure 5.**
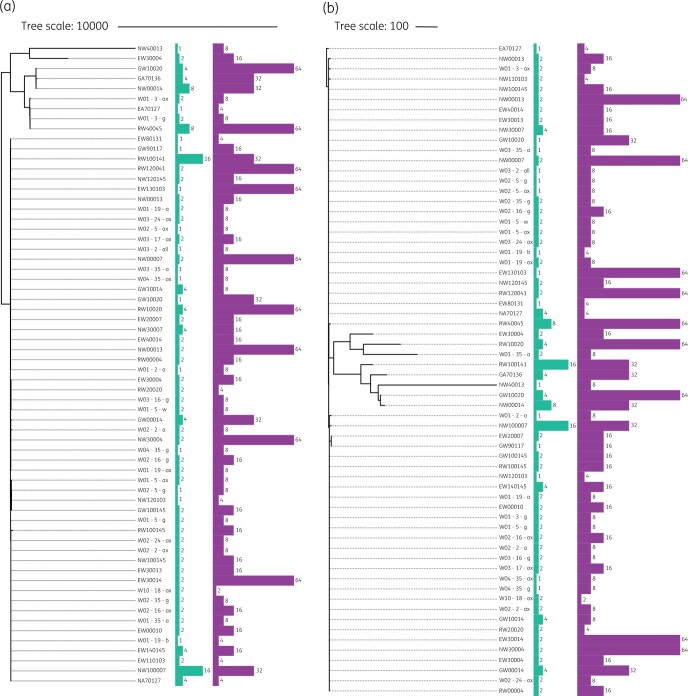
Phylogenetic tree of *S. epidermidis* based on (a) *norA* and (b) *norB* sequences and upstream regions and relation with octenidine (green) and chlorhexidine (purple) MICs (mg/L).

No relationship was detected between the presence of antimicrobial genes and elevated chlorhexidine or octenidine MICs (Figure 4 and Figure S2). There was no relationship between *qac* gene carriage and chlorhexidine and octenidine susceptibility. The *qac* genes were associated with lineage rather than antiseptic tolerance and were predominantly in *S. haemolyticus* isolates (*n = *48/49, 98.0%), and in 78.5% of *S. epidermidis* isolates (*n = *51/65). The chromosomal efflux systems, NorA and NorB have been implicated in antiseptic resistance in staphylococci with mutations in the promoter regions of both linked to elevated expression. Analysis of *norA* and *norB* genotypes did not reveal an association between genotype and antiseptic tolerance. Figure 5 shows phylogenetic trees for *S. epidermidis* isolates based on either *norA* or *norB* genotypes and associated antiseptic susceptibility (Figure 5).

## Discussion

We previously identified a correlation between increasing chlorhexidine usage and the emergence of MRSA isolates with reduced chlorhexidine susceptibility within one UK hospital trust, and we observed the emergence of strains with reduced susceptibility to octenidine after its introduction.^24^ Although the clinical implications of this are uncertain, in an outbreak of MRSA at an intensive care unit in London, Edgeworth *et al.*^41^ found the endemic clone demonstrated a 3-fold reduction in susceptibility to chlorhexidine. Widespread chlorhexidine use was believed to have contributed to the increased transmission of this strain in the unit.^41^ Antiseptic use in the NICU is of particular importance given the vulnerable patient population, and because agents are frequently used at lower concentrations than in adults owing to concerns about tolerability. There has been little focus on antiseptic resistance in neonatal care where lower concentrations of agents used may facilitate bacterial adaptation.^28^

Previous work has identified endemic strains that appear well adapted to the NICU environment. Well-adapted NICU strains are often characterized by high levels of antibiotic resistance and biofilm formation.^14^ One example of an NICU-endemic isolate is the globally disseminated *S. capitis* strain NRCS-A.^42^ An assessment of the phenotypic properties of this strain identified chlorhexidine tolerance as a contributor to its success within the NICU.^43^

The genome sequencing of our panel of NICU-derived isolates has offered some insight into the population structure of CoNS being carried by babies in the NICU. Most isolates were *S. epidermidis* or *S. haemolyticus*; previous studies investigating CoNS in the NICU have consistently identified *S. epidermidis* isolates more often than other CoNS, from both skin and gut-derived isolates.^14^^,^^44^ Analysis of the population structure of isolates and sites showed no significant difference between the UK and German units with highly related strains of the same sequence types present in both units (Figure 3). Therefore, there appear to be several related strains in circulation in both units, with no geographically dominant lineages seen.

Our findings showed isolates from the UK, where chlorhexidine has been extensively used for a long time, were less susceptible to chlorhexidine than those from the German unit where it has never been used. A large fraction of isolates from the UK had high MICs (greater than the 4 mg/L value proposed as a breakpoint for defining resistance) (Figure 1). We also found isolates taken upon admission to the UK unit were more susceptible to chlorhexidine than those taken subsequently (Figure 2). A similar finding has been noted previously with antimicrobial resistance, where CoNS isolates taken throughout a stay in NICU demonstrated more antimicrobial resistance than those taken at birth.^44^ Our findings support the hypothesis that entrants to the NICU rapidly acquire chlorhexidine-tolerant CoNS that are in circulation on the unit.

Isolates with decreased susceptibility to octenidine were more common in the UK, although this agent is not in routine use in this unit. All isolates with the highest octenidine MICs were from the UK (Figure 1). This suggests octenidine use may not readily select for decreased tolerance, as this was not seen in German isolates. Chlorhexidine–octenidine cross resistance appears unlikely as there was no correlation between susceptibility to the two agents (Figure S1). It is unclear why UK isolates were most tolerant to octenidine (in the absence of direct selective pressure) and relatively little is known about possible octenidine resistance mechanisms. As there was no significant difference in strain types between units, it is possible an unappreciated environmental factor or intervention (e.g. antibiotic usage), specific to the UK NICU, may impose a selective pressure impacting octenidine susceptibility.

The distribution of isolates with reduced susceptibility to chlorhexidine (and octenidine) across the phylogenies of all species studied, suggests chlorhexidine tolerance is not due to clonal expansion, but rather a phenotypic characteristic that could be acquired by multiple staphylococcal strains (Figure 4). This finding is consistent with our previous work with MRSA isolates and is highlighted by instances within all three phylogenies, where high and low MICs of chlorhexidine and octenidine were observed in closely related strains. Mechanistically, our findings may be accounted for by adaptations within the staphylococcal core genome.

Following whole genome sequencing we investigated the prevalence of known biocide tolerance genes but found no link between the presence of these genes and antiseptic susceptibility (Figure 4). The relationship between *qac* carriage and chlorhexidine and octenidine susceptibility has been the subject of debate.^45^ In this study, carriage of *qac* genes was associated with lineage rather than antiseptic susceptibility, a finding similar to that from our previous study of MRSA isolates and others.^24^ There was no difference in carriage of other AMR genes between isolates with high and low chlorhexidine MICs, and no association with carriage of accessory genes and antiseptic susceptibility (Figure S1 and Table S1).

Our previous study implicated changes in the NorA and NorB chromosomal efflux systems in chlorhexidine tolerance in MRSA. Changes to core chromosomal genes would explain the ability of all lineages to develop decreased antiseptic susceptibility, so we investigated the association between genotype for these alleles and phenotype (Figure 5). This included the promoter regions of both genes, but did we not identify a significant link. There are multiple regulatory loci known to influence expression of these systems that may be involved.^46^ We did analyse a set of closely related pairs of isolates where antiseptic tolerance differs, but did not identify any common changes in isolates with least susceptibility. This was complicated by the diversity of strain types and species present, in future, we aim to sequence all isolates to facilitate a genome-wide association study with sufficient power to identify genes underpinning our observations.

The relationship between NICU antiseptic use and antiseptic susceptibility has not previously been investigated in a prospective, multi-site surveillance study. Our findings demonstrate the potential of antiseptic use as a previously under-appreciated selection pressure within the NICU. The long-term use of chlorhexidine in the NICU may select for well-adapted strains, capable of growth at higher chlorhexidine concentrations, however the use of octenidine may be less likely to select for this. Further work is needed to understand the genetic mechanisms conferring these findings, and the clinical impact that may arise from carriage of isolates with antiseptic tolerance.

## Supplementary Material

dlab173_Supplementary_Data
