# Supplementary material for: Chlorhexidine gluconate usage is associated with antiseptic tolerance in staphylococci from the neonatal intensive care unit
Source: JAC Antimicrob Resist. 2021 Nov 17;3(4):dlab173. doi: 10.1093/jacamr/dlab173 (PMC8599896; doi:10.1093/jacamr/dlab173)
Supplement: dlab173_Supplementary_Data [file dlab173_supplementary_data.docx]

**Supplementary data**

**Figure S1.** **Relationship between susceptibility to CHG and OCT for all isolates. Linear regression (R^2^ = 0.04) performed using** **GraphPad Prism 7.**

**Table S1. Relationship between susceptibility to i) CHG ii) OCT and all antibiotics tested, for all isolates undergoing whole genome-sequencing.**

| **i) OCT** | Vancomycin: R^2^ = 0.018 | **ii) CHG** | Vancomycin: R^2^ = 0.004 |
| --- | --- | --- | --- |
|  | Gentamicin: R^2^ = 0.006 |  | Gentamicin: R^2^ = 0.014 |
|  | Benzylpenicillin: R^2^ = 0.023 |  | Benzylpenicillin: R^2^ = 0.002 |
|  | Cefotaxime: R^2^ = 0.012 |  | Cefotaxime: R^2^ = 0.022 |
|  | Ciprofloxacin: R^2^ = 0.001 |  | Ciprofloxacin: R^2^ = 0.031 |
|  | Daptomycin: R^2^ = 0.004 |  | Daptomycin: R^2^ = 0.016 |
|  | Fusidic acid: R^2^ = 0.004 |  | Fusidic acid: R^2^ = 0.021 |

**Figure S2. Carriage of AMR genes compared between isolates with low (red bars) and high (blue bars) MICs of chlorhexidine.**

**
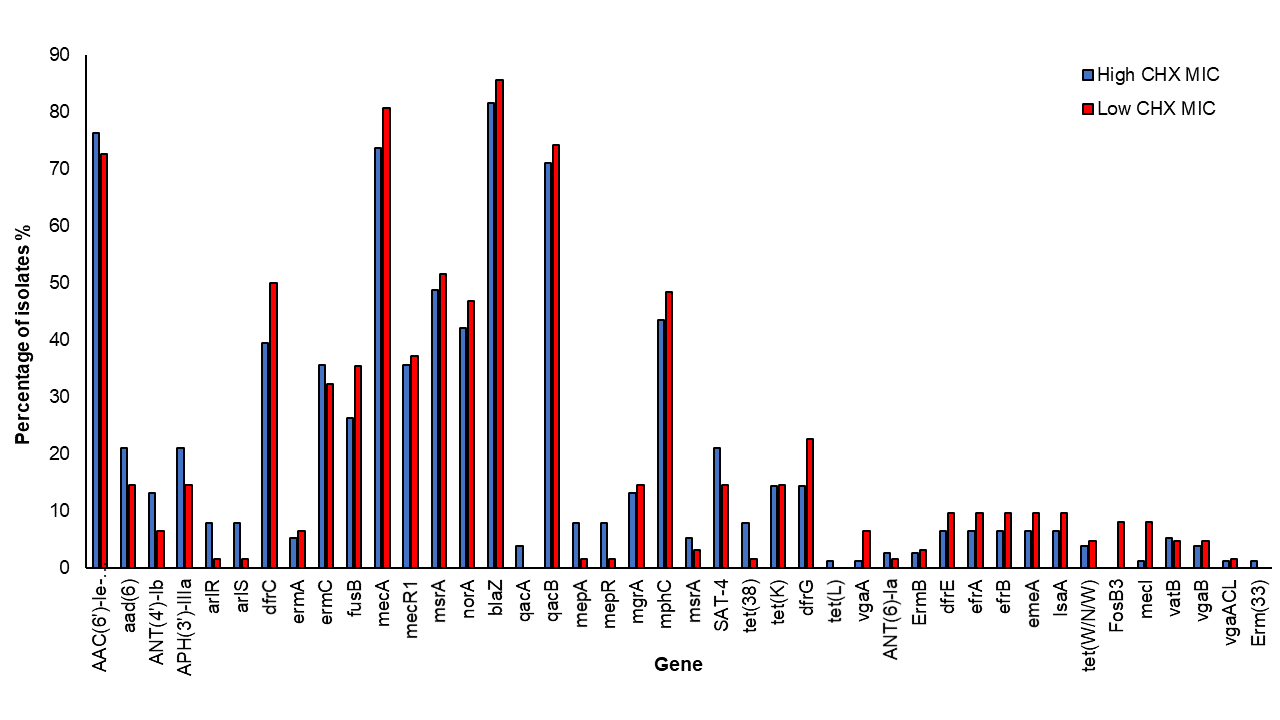
**
